# Dexamethasone in brain tumor patients: a real-world pharmacovigilance audit

**DOI:** 10.3389/fneur.2025.1632231

**Published:** 2025-08-20

**Authors:** Vindhya Prasad, Ali Elhag, Louis Onyiriuka, Engelbert Mthunzi, Sarah Hatch, Awais Naeem, Fatmahelzahraa Noureldin, Francesco Marchi, Ahmed Raslan, Yasir A. Chowdhury, Jonathan Shapey, Richard Gullan, Ranjeev Bhangoo, Francesco Vergani, Keyoumars Ashkan, Jose Pedro Lavrador

**Affiliations:** ^1^Neurosurgical Department, King’s College Hospital NHS Foundation Trust, London, United Kingdom; ^2^Service of Neurosurgery, Neurocenter of the Southern Switzerland, Regional Hospital of Lugano, Ente Ospedaliero Cantonale (EOC), Lugano, Switzerland; ^3^School of Biomedical Engineering & Imaging Sciences, King's College London, London, United Kingdom; ^4^Catolica Medical School, Oeiras, Portugal

**Keywords:** dexamethasone, brain tumors, steroid complications, neuro-oncology, pharmacovigilance

## Abstract

**Introduction:**

Dexamethasone is routinely prescribed for the management of peritumoral edema in brain tumor patients. Despite available orientations for its management in neuro-oncology patients, the individual needs according to the natural history of the disease and treatment options allied to a hierarchical system with multiple teams involved poses significant challenges in its real-world application.

**Methods:**

We conducted a retrospective single-centre observational study of 316 brain tumor referrals to a tertiary neurosurgical center over a 3-month period. Data was extracted from referral notes, multidisciplinary team (MDT) documentation and clinical records. Steroid-related variables such as indication, dose, duration, weaning plan, complications, and follow-up practices were collected alongside demographic and clinical data.

**Results:**

Of 316 referrals, 210 patients (66.5%) were started on steroids at baseline, yet only 6% had a documented weaning plan at that point. MDT referral occurred in 252 patients (79.7%), where steroid initiation was significantly associated with surgical management (*χ*^2^ = 13.1, *p* < 0.001). However, only 28.8% of MDT-referred patients had a documented steroid plan, with higher rates in surgical patients (41.3%) than those managed conservatively or with best supportive care (BSC) (16.5%, *p* < 0.001). Steroid-related complications occurred in 11.4% (24/210) of patients, most commonly wound infections. Prolonged steroid use (>2 weeks) (OR = 3.5, [95% CI: 1.1–11.0], *p* = 0.04), and absence of an MDT steroid plan (OR = 4.2, [95% CI: 1.2–15.0], *p* = 0.03) were significant predictors of complications, particularly of Common Terminology Criteria for Adverse Events (CTCAE) Grade 2–3 severity. Nurse-led clinic follow-up was more common in surgical patients (91%) than BSC patients (24.6%, *p* < 0.001) and supported steroid monitoring.

**Discussion:**

Prolonged steroid use and incomplete documentation of steroid plan were associated with increased steroid-related complications highlighting the need for more robust prescribing protocols and improved multidisciplinary follow-up.

## 1 Introduction

Dexamethasone, a glucocorticoid with minimal mineralocorticoid activity, has been the mainstay in managing brain tumor patients for decades since its first description in literature in 1961 (1). It has shown to act by reducing vasogenic oedema and alleviating elevated intracranial pressure, improving symptoms as early as 12 to 24 h after initiation (2). These rapid effects underscore the drug’s efficacy in reducing brain edema and support its strategic use in subacute cases. Furthermore, when combined with hyperosmolar therapy, dexamethasone may offer enhanced benefits in the management of more acute presentations of intracranial hypertension (3–5). There is also some evidence of potential antitumor effect through inhibition of tumor growth, promotion of oncolysis and modulation of the tumor microenvironment (6).

However, dexamethasone has significant side effects, especially with prolonged use (7). Common complications include hyperglycemia, steroid-induced diabetes, muscle weakness, osteoporosis, and increased infection risk due to immunosuppression. Neuropsychiatric effects such as mood disturbances and steroid-induced psychosis occur in approximately 5–10% of patients, while gastrointestinal complications, including ulcers and gastrointestinal bleeding, are reported in 2–6% of cases (7). Moreover, prolonged corticosteroid therapy can suppress the hypothalamic–pituitary–adrenal (HPA) axis, with studies indicating that adrenal insufficiency may develop in 40–60% of patients after extended use (7). Higher doses were linked to poorer overall survival and greater toxicity as indicated by multiple large-scale studies: Mistry et al. reported higher rates of steroid dependence, infection, and reduced survival following postoperative dexamethasone use in glioblastoma (8); Wasilewski et al. found that cumulative doses ≥122 mg were independently linked to worse survival in patients undergoing brain metastasis resection (9); and Cho et al. identified dexamethasone doses ≥100 mg around radiosurgery as an independent predictor of mortality, despite advances in systemic therapy (10).

There is considerable variability in dexamethasone dosing, treatment duration, and tapering strategies among neurosurgeons, with no standardized consensus on the optimal initial dose, maintenance regimen, or weaning protocols (11). Given the complex referral pathways in neuro-oncology, patients are often assessed at multiple centers before reaching specialized units, creating potential gaps in communication and treatment continuity. Inconsistent documentation and misinterpretation of treatment plans can lead to suboptimal steroid prescribing, particularly regarding dosing, duration, and tapering. Establishing clear, standardized guidance for steroid use and ensuring proper follow-up of treatment recommendations from specialized centers is essential to improving patient outcomes. This study provides real-world data on the practical application and pharmacovigilance of steroids in brain tumor management, identifying areas where the current practice may diverge from best evidence and highlighting opportunities for improved oversight.

## 2 Methods and materials

### 2.1 Data collection and definition of variables

**Figure 1:**
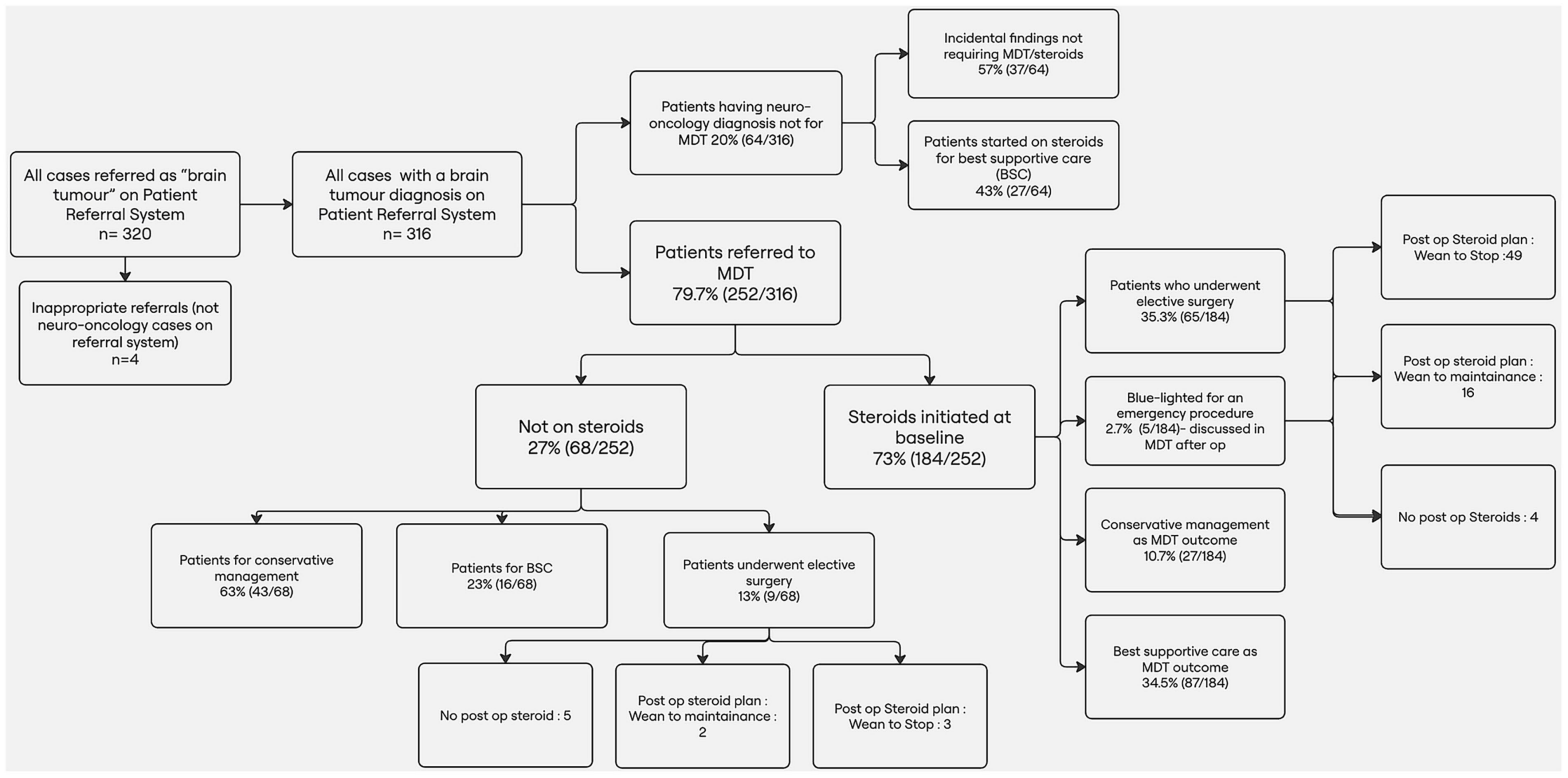
Flow diagram summarizing the methodology and results from initial referral to MDT outcomes and steroid management.

A retrospective single center cohort study, authorized upon review by the governance neurosurgical team (Audit number: NS202402), was conducted reviewing all cases referred to as “brain tumor” in the patient referral system over a 3-month period (1st December 2023–29th February 2024) to assess compliance with the pharmacovigilance and clinical use of steroid prescriptions. This is summarized in Figure 1.

The Patient Content Store (PCS) system is the electronic referral platform used for all acute neurosurgical referrals to our center. It serves as the central referral hub for neurosurgical care across our catchment area, facilitating streamlined access to specialist services. After excluding inappropriately tagged referrals with non-neuro-oncological diagnoses (e.g., abscesses and vascular pathologies), all remaining patients with confirmed neuro-oncological diagnoses were screened for details of initial steroid management.

**Figure 2:**
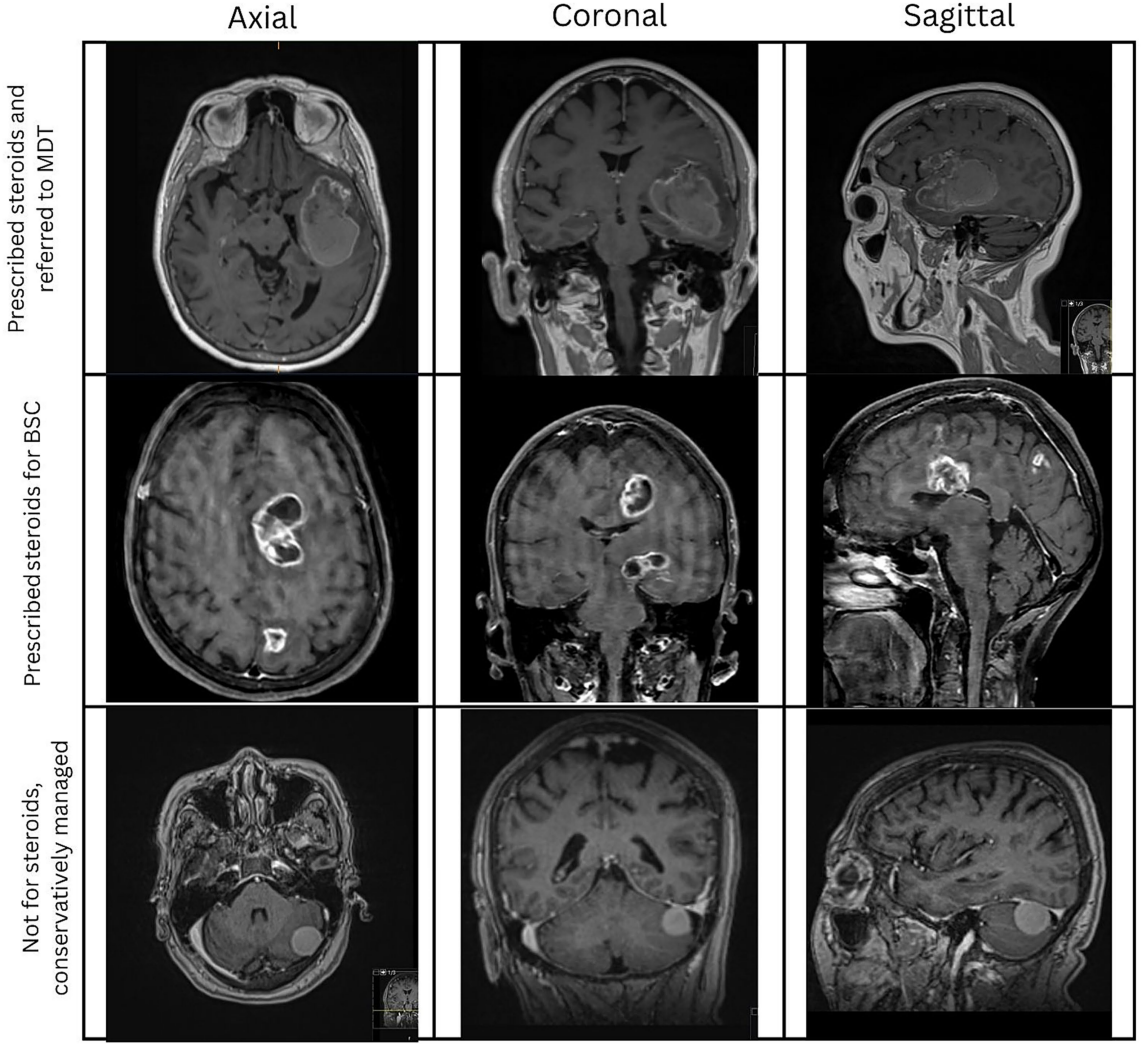
Rationale of steroid prescription and MDT referral as initial management by on-call neurosurgery based on initial referral scan.

This included assessment of:

- Appropriateness of Steroid Indication was determined based on the presence of clinical features suggestive of raised intracranial pressure (e.g., headache, nausea, altered consciousness) and/or radiological evidence of mass effect (midline shift and ipsilateral effacement of sulci) or vasogenic edema on neuroimaging. Figure 2 demonstrates radiological rationale for steroid initiation and MDT referral decisions by on-call neurosurgery based on initial referral imaging.
- Completeness of Steroid Plan Documentation: If the suggested plan included the prescribed dose and duration, the presence of a weaning regimen (dose and duration) and proton pump inhibitor (PPI) co-prescription and is formally documented in the electronic referral portal as the outcome of the acute referral.
- Outcome of the Acute Referral: All acute referrals were categorized based on their subsequent management trajectory. Patients were either referred to the multidisciplinary team (MDT) for further discussion, admitted urgently to the neurosurgical service, managed conservatively, or recommended best supportive care (BSC) without further escalation.

For patients where the outcome from the acute referral was “referral to neuro-oncology MDT,” electronic health records were reviewed to obtain MDT recommendations on treatment and further steroid management plans. For patients who underwent surgery (resection or biopsy), their perioperative steroid treatment plans were assessed. For those who did not undergo surgery (conservatively managed or best supportive care), medical records were reviewed for available steroid treatment plans. The presence of complications related to steroid treatment and their management was retrieved from patient electronic records for both groups. The classification of a clinical event as a steroid-related complication was performed by the clinical team assessing the patient at that time and not retrospectively upon clinical documentation consultation during this audit to avoid attribution bias by the team involved in data collection in the present audit (Figure 1). A 2-week treatment period cut-off was used to assess the impact of steroid exposure and steroid-related complications after a relevant literature review (7, 12).

Using the Common Terminology Criteria for Adverse Events (CTCAE) classification, complications were graded based on severity (13). Most were classified as Grade 2 (Moderate) or Grade 3 (Severe). For example, wound infections, which required medical or surgical intervention and hospitalization, were graded as Grade 3. Gastrointestinal complications, psychosis, and systemic infections were also classified as Grade 3, given their potential to impair daily function or necessitate hospital care. Conversely, weight gain, sleep disturbances, and Cushingoid features were categorized as Grade 1 or 2, as they impacted quality of life but did not usually require acute intervention. Notably, steroid-induced diabetes and osteoporosis, while often managed outpatient, were considered Grade 2–3 depending on functional impact and required treatment.

### 2.2 Statistical analysis

Statistical analysis was performed using Python (version 3.10), with packages including pandas, scipy, and statsmodels. Descriptive statistics were used to summarize patient demographics, steroid usage patterns, and complication rates. Group comparisons were conducted using independent two-sample t-tests for continuous variables and chi-square tests (or Fisher’s exact test where appropriate) for categorical variables. Logistic regression modeling was applied to identify predictors of steroid-related complications. Univariate significant variables and initial dose, duration of steroid therapy, and documentation of MDT steroid plans were added as covariates. Odds ratios (ORs) with 95% confidence intervals were reported. A *p*-value of <0.05 was considered statistically significant. All statistical tests were two-tailed.

## 3 Results

Out of 320 acute referrals, 316 patients (98.75%) with neuro-oncological diagnoses were included in the analysis (3 abscesses; 1 cavernoma excluded). The cohort had equal sex distribution (1:1) with a mean age of 66.3 ± 15.8 years. The most common presenting symptoms were focal neurological deficits (25.9%) and headaches (24.1%). Brain metastases were the most frequent diagnosis (47.8%, *n* = 151), followed by high-grade gliomas (18.0%, *n* = 57), meningiomas (11.1%, *n* = 35), and low-grade gliomas or other tumors (6.3% each, *n* = 20). Most tumors were supratentorial (84.5%) and intra-axial (69.9%). Right-sided lesions (46.2%) were slightly more common than left-sided lesions (40.8%). Tables 1, 2 summarize the population characteristics and tumor characteristics.

**Table 1:** Basic characteristics of study population and tumor location.

Variable	No steroid – related complications (*n* = 295)	With steroid- related complications (*n* = 20)	*p*-value
Age (mean ± SD)	65.0 ± 15.2	56.5 ± 17.5	0.046*
Sex (Male %)	50.5%	40.0%	0.279
Comorbidities (%)*	63.7%	75.0%	0.230
Presenting symptom (%)	Neurology: 26.1%, Headache: 24.1%, Seizures: 13.6%, Other: 36.2%	Similar distribution	0.555
Laterality (%)	Right: 45.8%, Left: 41.0%, Bilateral: 13.2%	Similar distribution	0.134
Axial location (%)	Intra-axial: 70.0%, Extra-axial: 29.7%, Mixed: 0.3%	Similar distribution	0.866

^*^Paired *t* test for univariate analysis.

**Table 2:** Tumor histology in steroid initiation cohort.

Initial diagnosis	Total patients on steroids	With steroid related complications (*n*, %)	No steroid related complications (*n*, %)
High-Grade Glioma (HGG)	80	10 (12.5%)	70 (87.5%)
Metastatic tumor	85	8 (9.4%)	77 (90.6%)
Meningioma	25	2 (8.0%)	23 (92.0%)
Other (e.g., LGG, other)	20	1 (9.1%)	19 (90.9%)
Total	210	21 (10%)	189 (90%)

### 3.1 Acute referral outcomes

Of 316 patients, 210(66.4%) were started on steroids at referral by the neurosurgical team and local hospital. Only 13/210 (6%) had a documented weaning plan at this stage. 64 patients (20%) were not referred to the MDT. Of these, 37 (57%) were managed conservatively without steroids, and 27 (43%) received steroids for best supportive care (BSC) - 4/27 (14.8%) had documented steroid plans (Figure 1).

A total of 252 patients (79.7%) were discussed at the MDT. Of these, 184 (73%) had already been started on steroids. MDT outcomes for these included BSC in 87 patients (34.5%), surgical resection or biopsy in 70 (27.7%), and conservative management in 27 (10.7%). Among the 68 patients not on steroids prior to MDT, 43 were managed conservatively; 16 received BSC, and 9 underwent surgery.

Among the 79 patients who underwent surgery, 70 (88.6%) received post-operative steroids; 52 (74.3%) were planned to wean to cessation and 18 (25.7%) to maintenance. Patients initiated on steroids were significantly more likely to undergo surgery than those not started on steroids (*χ*^2^ = 13.14, *p* = 0.00029).

### 3.2 Steroid plans and regimens

There was a statistically significant association between steroid initiation and MDT outcome (*χ*^2^ = 13.14, *p* = 0.00029). Patients started on steroids were more often recommended for surgery (70 vs. 9), while those not on steroids were more frequently managed with BSC (59 vs. 114). This suggests that early steroid use may reflect clinical severity influencing MDT decisions.

Steroid documentation remained variable. Only 53 of 184 patients (28.8%) had a clear steroid plan documented by the MDT. Surgical patients were more than twice as likely to receive such guidance compared to BSC patients (41.3% vs. 16.5%, *χ*^2^ = 13.6, *p* = 0.0002). Among patients initiated on steroids, the most common regimen was 4 mg twice daily (36.6%), followed by an 8 mg loading dose plus 4 mg twice daily (20.7%). Surgical patients were more frequently started on the lower regimen (40.5%), while BSC patients more commonly received the higher loading dose (28.1%). This difference was statistically significant (*χ*^2^ = 6.38, *p* = 0.04), suggesting dosing practices were tailored to perceived disease burden or prognosis.

### 3.3 Steroid-related complications

**Figure 3:**
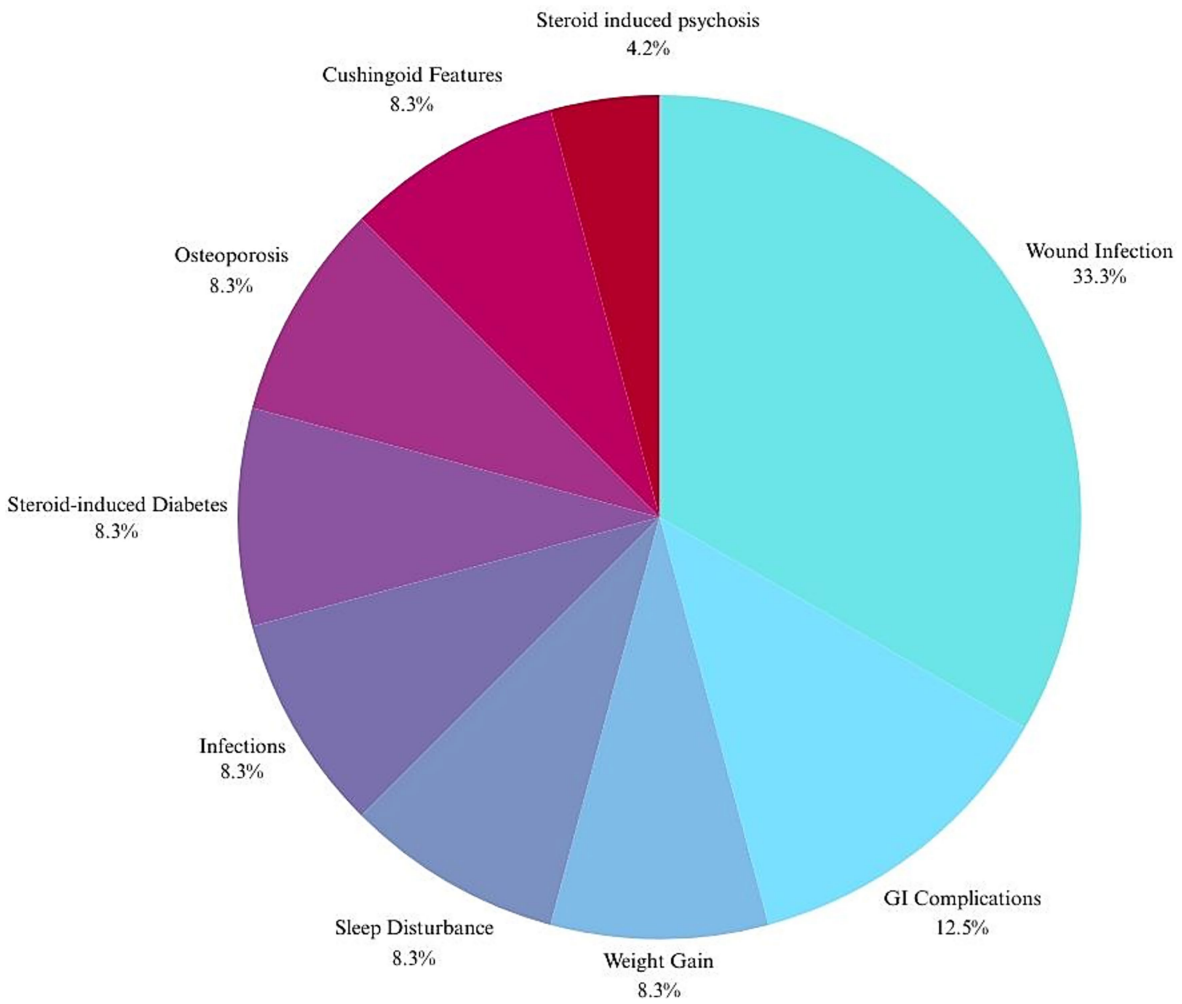
Steroid-related complications occurred in 24 of 210 patients (11.4%): Wound infections (8/24, 33.3%), GI complications in 3/24 (12.5%), weight gain 2/24 (8.3%), sleep disturbance 2/24 (8.3%), infections 2/24 (8.3%), diabetes 2/24 (8.3%), osteoporosis 2/24 (8.3%), Cushingoid features 2/24 (8.3%) and psychosis in 1/24 (4.2%).

Steroid-related complications occurred in 24 of 210 patients (11.4%), most commonly wound infections (8/24, 33.3%). Other complications (each in 2 patients, 8.3%) included weight gain, sleep disturbance, infections, diabetes, osteoporosis, and Cushingoid features. GI complications were reported in 3 patients (12.5%); psychosis in one (4.2%) (Figure 3). Table 3 describes the distribution of complications between the two management groups and classifies the complications into CTCAE grades.

**Table 3:** Distribution of steroid-associated complications by management group (surgical vs. BSC/conservative).

Complications (*n* = 24)	Management		Common Terminology Criteria for Adverse Events (CTCAE)
	Surgical cohort (*n* = 13)	Best supportive care/conservative cohort (*n* = 11)	
Wound infection	8 (61.0)	0 (0.0)	Grade 3
Gastro-intestinal complications	1 (7.6)	2 (18.1)	Grade 2
Weight gain	0 (0.0)	2 (18.1)	Grade 1
Sleep disturbance	0 (0.0)	2 (18.1)	Grade 1
Systemic infections	1 (7.6)	1 (9.0)	Grade 3
Steroid induced diabetes	0 (0.0)	2 (18.1)	Grade 2
Osteoporosis	0 (0.0)	2 (18.1)	Grade 2
Cushingoid features	1 (7.6)	1 (9.0)	Grade 2
Psychosis	1 (0.0)	0 (0.0)	Grade 3

Surgical management was significantly associated with higher-grade complications. All wound infections (n = 8) were observed exclusively in surgical patients and classified as Grade 3 under CTCAE. Surgical patients accounted for 10 of the 11 Grade 3 complications, and this association between surgery and Grade 3 adverse events was statistically significant (*χ*^2^ = 8.28, *p* = 0.018) (Table 4).

**Table 4:** Association between CTCAE complication grades and risk factors.

Predictor	CTCAE Grade 1	CTCAE Grade 2	CTCAE Grade 3
> 2 weeks on steroids	0.16	0.002*	0.006*
Lack of MDT steroid plan	0.56	0.08	0.01*
Surgical management	–	0.92	0.018*

Chi-square *p*-values for the association of each risk factor (prolonged steroid use, lack of MDT plan, and surgical management) with CTCAE Grade 1, 2, and 3 complications. Asterisks (*) denote statistical significance (*p* < 0.05), we had no grade 1 complications in the surgical group.

Mean steroid duration was 18 ± 10 days (median: 14; range: 4–60). Roughly 35–40% of patients, and 38% of surgical patients, were on steroids for >2 weeks. Initial steroid dose was not significantly associated with complications (8.2 mg/day vs. 7.6 mg/day; *p* = 0.20). Among patients with complications, 13/24 (54%) had an MDT steroid plan documented vs. 11/24 (46%) who did not.

Patients who underwent surgery had a higher complication rate than non-surgical patients due to post-operative wound infections (33.3%). However, this did not prove to be a statistically significant predictor of complications (15.2% vs. 7.4%, *χ*^2^ = 3.1, *p* = 0.08). Histology was also not associated with complication rates (*χ*^2^ = 0.57, *p* = 0.90).

In logistic regression, duration of steroid use (OR = 3.5, [95% CI: 1.1–11.0], *p* = 0.04) and absence of an MDT steroid plan (OR = 4.2, [95% CI: 1.2–15.0], *p* = 0.03) were significant predictors of complications. Initial dose was not (*p* > 0.1) (Table 5).

**Table 5:** Regression analysis of adverse effect predictors.

Predictor	Odds ratio (OR)	95% CI	*p*-value
> 2 weeks on steroids	3.5	1.1–11.0	0.04*
Lack of MDT steroid plan	4.2	1.2–15.0	0.03*
High initial steroid dose (≥8 mg)	1.4	0.5–4.0	0.50
Histological diagnosis	≈1.0	–	0.6
Surgical management	1.3	0.5–3.6	0.40
Age (per year)	0.97	0.93–1.01	0.16

Asterisks (*) Denote statistical significance (*p* < 0.05).

When stratified by CTCAE grade, prolonged steroid use beyond 2 weeks was significantly associated with both Grade 2 (*p* = 0.002) and Grade 3 (*p* = 0.006) complications, but not with Grade 1 (*p* = 0.16). Similarly, lack of an MDT steroid plan was significantly linked to Grade 3 complications (*p* = 0.01) and showed a trend for Grade 2 (*p* = 0.08) but was not associated with Grade 1 toxicity (*p* = 0.56). These findings further underscore the disproportionate impact of poor planning and extended duration on moderate to severe adverse events (Table 4).

NLC follow-up was significantly more common in surgical patients (72/79, 91%) than BSC patients (28/114, 24.6%) (*χ*^2^ = 84.0, *p* < 0.001) who were started on steroids. Average follow-up occurred at 12 ± 4 days. Half of the 24 patients who returned with complications (*n* = 12) were seen at NLC. Among the surgical patients followed up in NLC, 11/72 (15.3%) developed complications (8 were wound infections), and 1/28 of BSC patients (3.6%) had NLC follow-up. The remaining 12 patients were started on long-term steroids for BSC but were not followed up in NLC (*χ*^2^ = 2.54, *p* = 0.11).

## 4 Discussion

This study provides real-world insights into the prescribing patterns, documentation practices, and complication rates associated with corticosteroid use in brain tumor patients across a tertiary referral network. Of the 316 patients analyzed, 66.5% (210/316) were initiated on steroids at the time of referral; however, only 6% (13/210) had a documented weaning plan at that stage. MDT involvement significantly influenced this variability. Patients referred to the MDT were more likely to be started on steroids (73%) and receive structured weaning guidance (28.8%) compared to those not discussed in MDTs (42.2% started steroids, with only 14.8% having documented plans). 11.4% of patients who started on steroids experienced related complications, one third of them being wound infections, related to the length of steroid treatment and lack of MDT documented plan.

A key finding was the relationship between early steroid initiation and MDT treatment decisions. Patients started on steroids at referral were significantly more likely to be recommended for surgical intervention, while those not on steroids were predominantly assigned to best supportive care (BSC) (*χ*^2^ = 13.14, *p* = 0.00029). These data may reflect a dual bias: patients who require surgery are more likely to have a more significant mass effect from the tumor and therefore requiring steroids from initial presentation; and/or in patients considered for the BSC pathway, clinicians want to avoid unnecessary steroid treatment given the potential complications and side-effects of long-term treatment. Additionally, documentation was more frequent in surgical patients (41.3%) than those managed conservatively or with BSC (16.5%) (*χ*^2^ = 13.6, *p* = 0.0002), highlighting treatment-pathway-based disparities in steroid oversight. This finding reflects an asymmetry in patient care between surgical and non-surgical care that must be addressed on a wider scale. As highlighted by other groups, there is a need to streamline the acute assessment of patients with brain tumors (14). Although surgical patients typically require greater MDT involvement due to ongoing treatment decisions, our data suggest that patients on the BSC pathway may equally benefit from integrated care and structured follow-up.

Palliation is often perceived at a later stage of treatment in patients who have undergone surgery and oncology treatment. However, patients on the BSC pathway require early input as well, since the overarching goals of symptom relief and preservation of quality of life remain the same (15). Particularly, steroid management is crucial as these patients are likely to be dependent on this medication for longer periods and, therefore, more prone to complications related to it. BSC-focused MDTs or clinics should be considered to address this unmet need in the bigger picture of the neuro-oncology population referred to a tertiary neuro-oncology service.

Although the most common regimen at referral was 4 mg twice daily (36.6%), patients in the BSC group were more often prescribed an 8 mg loading dose followed by 4 mg BD (28.1%), likely reflecting greater symptom burden (*χ*^2^ = 6.38, *p* = 0.04). However, the initial dose was not predictive of complications (*p* = 0.20), consistent with existing literature (7, 16, 17). Instead, the duration of corticosteroid therapy emerged as a key predictor. Patients on steroids for more than 2 weeks had 3.5 times the odds of developing adverse effects (OR = 3.5, [95% CI: 1.1–11.0], *p* = 0.04), particularly Grade 2 (*p* = 0.003) and 3 (*p* = 0.006). Dietrich et al. extensively described how complications such as hyperglycemia, muscle wasting, osteoporosis, and neuropsychiatric disturbances are strongly correlated with extended exposure, especially beyond 2–3 weeks (7). Similarly, Hempen et al. emphasized that even standard doses become harmful over time due to the systemic metabolic effects of corticosteroids (16). Thus, our findings reinforce the clinical imperative to minimize treatment duration and prioritize early weaning strategies, particularly in patients managed conservatively or without a clear endpoint for steroid discontinuation.

These considerations are essential for MDT discussions, as they support treatment prioritization for patients on steroids and highlight the need for broader multidisciplinary involvement, such as regular input from endocrinology teams, to optimize steroid-related management (18). Our results do not show cases of steroid withdrawal syndrome, which resembles adrenal insufficiency in the presence of a working hypophysis-pituitary–adrenal axis of patients with true adrenal insufficiency due to inadequate steroid weaning. Even though continuous steroid use during treatment (chemotherapy and/or radiotherapy) is possibly one of the reasons, there is a potential bias toward lack of diagnosis due to endocrinology involvement restricted to tertiary centers and the latency implicit in some of these complications which make them suitable for diagnosis in late-effects clinic only which was not captured in this project (19, 20). This further emphasizes the role of a steroid-management team within a broader neuro-oncology MDT concept.

Furthermore, absence of a documented steroid plan at MDT quadrupled the overall risk of complications (OR = 4.2, 95% CI: 1.2–15.0, *p* = 0.03) and was also associated with a significantly higher risk of developing more severe (Grade 3) adverse events (*p* = 0.03), reinforcing the critical role of structured prescribing practices. As emphasized in national guidelines, the primary role of the multidisciplinary team (MDT) is to provide diagnostic clarification and high-level treatment recommendations, particularly regarding surgical candidacy and oncological planning (2, 21, 22). However, detailed and adaptive medication plans, especially for tapering symptomatic therapies such as corticosteroids, are more appropriately developed by the local clinical team, who are responsible for the patient’s longitudinal follow-up and are best positioned to monitor evolving clinical needs (7). Since patients are not routinely re-discussed at the MDT after the initial treatment decision (particularly if BSC or follow-up in clinic is the outcome), the absence of formalized corticosteroid weaning plans in MDT documentation may indicate an inherent limitation in MDT workflows rather than an oversight. Therefore, responsibility for ongoing steroid optimization falls to the treating teams who maintain regular contact with the patient.

In our cohort, patients who received follow-up through a nurse-led clinic had more consistent documentation of steroid plans and dose modifications, and it emerged as a valuable tool for post- steroid surveillance. Patients followed up in NLC were more likely to have documented plans and experienced lower complication rates (8% vs. 13%), although this trend was not statistically significant. Among BSC patients, 92.3% of those who developed complications were not followed up in NLC, underlining a potential missed opportunity for mitigation. Previous studies have demonstrated the same (7, 11). This suggests that structured nurse-led follow-up not only complements MDT decision-making but may also reduce adverse effects through better continuity of care and enhanced steroid stewardship.

Aligning with existing guidelines, this study also advocates for judicious use of corticosteroids, reserving them for patients with symptomatic peritumoral oedema (12, 21–25). Given the structure of neuro-oncology services in the United Kingdom, where MDTs offer high-level recommendations and local teams manage day-to-day care, pharmacovigilance can be improved through targeted system-level changes. As MDTs cannot reliably implement detailed tapering plans, integrating structured steroid weaning templates into MDT documentation would prompt more consistent local action. Our findings also support routine referral to nurse-led clinics for patients discharged on steroids, especially those lacking documented plans, to improve monitoring and adherence. Finally, standardized institutional protocols, aligned with international guidelines, would promote consistency across teams, and reduce variation in steroid prescribing.

Some limitations can be identified in this audit. Firstly, its retrospective design may be subject to documentation bias, relying on the accuracy and completeness of clinical notes. For example, systematic baseline performance status was not recorded as this was not a mandatory information at the time of the acute neurosurgery referral, which limits its use in the analysis performed despite the literature supporting its relevance (17, 23, 24). Therefore, we used neurological deficits at presentation as a proxy/surrogate for some of the information that can be transmitted via the overall performance status. Tumor volumetric data at the time of steroid decision-making were unavailable, as most patients were initially referred via the acute neurosurgical portal with only a CT scan. This significantly limited volumetric assessment, particularly for intra-axial lesions, where MRI provides greater accuracy. Therefore, this information was not used for analysis. However, the fact that the steroid decision was performed without a formal volumetric assessment supports its absence from the multivariate analysis models. Secondly, while complications such as infections were attributed to steroid use, we acknowledge that these may have multifactorial etiologies, including tumor burden, comorbidities, and perioperative factors. Nonetheless, given the well-established immunosuppressive effects of corticosteroids, such complications were conservatively considered as steroid-related for the purpose of analysis. Additionally, the lack of uniform follow-up intervals may have led to underreporting late complications or inconsistencies in documentation of tapering plans. Lastly, data from nurse-led clinics were limited to documented notes, and some follow-up activities may have gone unrecorded.

This study offers several strengths, including its real-world scope, encompassing a large cohort of brain tumor patients referred to a UK tertiary neuro-oncology center. It provides granular data on steroid prescribing practices, documentation trends, and complications, with stratification by referral pathway and MDT outcome. The inclusion of both surgical and non-surgical patients reflects the full clinical spectrum, allowing for generalizable insights into practice variation and pharmacovigilance challenges.

## 5 Conclusion

The complex, multilayered nature of neuro-oncology referral systems, coupled with the variability in individual patient needs, presents substantial challenges to the consistent application of evidence-based steroid prescribing guidelines. Our findings demonstrate that prolonged corticosteroid use beyond 2 weeks and the absence of a clearly documented steroid plan at MDT level are both significantly associated with increased complication rates. To mitigate these risks, enhanced pharmacovigilance is essential. This includes improved documentation of steroid recommendations at the point of MDT discussion, routine referrals to nurse-led follow-up clinics, and proactive engagement of local treating teams in adapting steroid regimens during longitudinal care. Such integrated, multidisciplinary approaches are necessary to preserve the therapeutic benefits of steroids while minimizing preventable adverse effects in brain tumor patients.

## Data Availability

The raw data supporting the conclusions of this article will be made available by the authors, without undue reservation.
